# The N-Terminal α-Helix of Potato Virus X-Encoded RNA-Dependent RNA Polymerase Is Required for Membrane Association and Multimerization

**DOI:** 10.3390/v14091907

**Published:** 2022-08-28

**Authors:** Xue Jiang, Yameng Luan, Mengzhu Chai, Yingshuai Yang, Yuting Wang, Wenjia Deng, Yonggang Li, Xiaofei Cheng, Xiaoyun Wu

**Affiliations:** 1 College of Agriculture, Northeast Agricultural University, Harbin 150030, China; 2 Key Laboratory of Germplasm Enhancement, Physiology and Ecology of Food Crops in Cold Region of Chinese Education Ministry, Northeast Agricultural University, Harbin 150030, China

**Keywords:** potato virus X, membrane association, methyltransferase, multimerization, RNA-dependent RNA polymerase

## Abstract

Positive-sense single-stranded RNA viruses replicate in virus-induced membranous organelles for maximum efficiency and immune escaping. The replication of potato virus X (PVX) takes place on the endoplasmic reticulum (ER); however, how PVX-encoded RNA-dependent RNA polymerase (RdRp) is associated with the ER is still unknown. A proline-kinked amphipathic α-helix was recently found in the MET domain of RdRp. In this study, we further illustrate that the first α-helix of the MET domain is also required for ER association. Moreover, we found that the MET domain forms multimers on ER and the first α-helix is essential for multimerization. These results suggest that the RdRp of PVX adopts more than one hydrophobic motif for membrane association and for multimerization.

## 1. Introduction

The replication of positive-sense single-stranded RNA (+ssRNA) viruses usually takes place in virus-induced membranous structures called viral replication organelles (VROs) [1,2]. VROs are confined membranous compartments that are remodeled by viral replication-associated proteins from the endomembrane, e.g., the endoplasmic reticulum (ER) and the outer membrane of endosomes, mitochondria, peroxisomes, and chloroplasts [3,4,5,6]. VROs provide not only a suitable niche to concentrate essential viral and host factors for efficient viral RNA synthesis, but also a protective environment to the double-stranded RNA (dsRNA) replication intermediates from cellular dsRNA nucleases and sensors, e.g., the toll-like receptor 3 (TLR3), RNA helicase RIG-I, MDA-5, dsRNA-dependent protein kinase R (PKR), and Dicer (or Dicer-like, DCL) [7,8,9,10,11]. Viral replication-associated proteins of some +ssRNA viruses, e.g., protein A of *flock house virus* (FHV), NSP3 of coronaviruses, and NS5B of *hepatitis C virus* (HCV), interact with the endomembrane via a transmembrane domain [12,13,14]; however, replication-associated proteins of many other +ssRNA viruses, such as nsP1 of *chikungunya virus* (CHIKV) and 1a of *bromo mosaic virus* (BMV), are peripherally associated with the membrane through one or more amphipathic motifs (α-helix or loop) [15,16,17]. Electron cryo-electron microscope (cryo-EM) studies have shown that viral membrane-associated proteins can form crown-shaped complexes with a pore in the middle for the import of replication materials and the export of progeny RNA into the cytoplasm [14,15,18,19,20,21,22].

The genus *Potexvirus* of the family *Alphaflexviridae* contains a group of filamentous +ssRNA viruses, which infect a large group of crops and ornamental plants. The genome of PVX is a +ssRNA molecule of about 6.4 kilobases (kb) in size that contains five open reading frames (ORF1-5). ORF1, located at the 5′-proximal region of the genome, encodes the viral RNA-dependent RNA polymerase (RdRp) [23]. RdRp is a multi-domain protein that processes the N-terminal methyltransferase domain (MET), central RNA helicase (HEL), and C-terminal replicase domain (REP). ORF2-4 are partially overlapped and comprise the so-called triple gene block (TGB), and encode TGBp1, TGBp2, and TGBp3, respectively [24]. ORF5 is located on the 3′-proximal region of the genome encoding the viral coat protein (CP) [25]. Studies have shown that RdRp is the only viral protein that is absolutely required for viral replication, while TGBp1-3 and CP are required for viral cell-to-cell or long-distance movement [26,27]. Recently, we found that TGBp2 was also co-localized with RdRp to enhance viral replication and cell-to-cell movement [28]. The replication of potexviruses is also associated with the endomembrane [29]. For instance, the RdRp of *potato virus X* (PVX), *bamboo mosaic virus* (BaMV), and *plantago asiatica mosaic virus* (PlAMV) locates to the ER as granules during virus infection [30,31,32]. A proline-kinked amphipathic α-helix at the C-terminal of the MET domain of PlAMV was recently found to be required for its membrane association [31]. Previously, we found that the N-terminal part of the PVX MET domain, excluding the proline-kinked amphipathic α-helix, also behaved as a membrane protein [28], suggesting that there are additional membrane-association motifs in the MET domain. In this study, we further analyzed the membrane association of the PVX-encoded MET domain through biochemical and cell biological methods. Our results showed that the first α-helix is required both for membrane-association and multimerization.

## 2. Materials and Methods

### 2.1. Plant Growth Conditions

*Nicotiana benthamiana* seedlings were grown in Sunshine^®^ Mix #1/Fafard^®^-1P (Sun Gro Horticulture, Agawam, MA, USA) in a plant growth chamber at 23 °C with a 16:8 (light:dark) photoperiod and ~60% humidity. Seedlings were watered every other day and nourished once a week.

### 2.2. Vector Construction

Coding sequences of the MET domain were amplified with the Phanta^®^ Super Fidelity DNA Polymerase (Vazyme, Nanjing, China) using pGR107 as the template. Amplified fragments were inserted into a modified entry vector pDONR207 (pDONR207m) by homologous recombination. The resulting entry clone was transferred into pEarley101 to construct C-terminal yellow fluorescent protein (YFP)-tagged constructs by the Gateway LR Clonase II enzyme mix (Invitrogen, Shanghai, China). To construct a 10×Histidine-tagged MET, the coding region of 10×Histidine was included in one primer to amplify the MET 1-416 aa coding region; the amplified fragment was used to replace the *ccdB* cassette and YFP-coding region in pEarley101 via recombination. All primers used in this study are listed in Supplementary Table S1. All plasmids were confirmed by Sanger sequencing.

### 2.3. Transient Expression

Transient expression on *N. benthamiana* leaves was performed as previously described [28]. In brief, the recombinant binary construct was introduced into the *Agrobacterium rhizogenes* strain GV3101 expressing p19 of *tomato bushy stunt virus* (TBSV) by electroporation. Transformants were screened with PCR and a positive clone was grown in a liquid Luria-Bertani (LB) medium supplied with suitable antibiotics overnight at 28 °C. *A. rhizogenes* was collected by centrifugation at room temperature and washed twice by an infiltration buffer [10 mM 4-morpholineethane sulfonic acid (MES), PH 5.6; 10 mM MgCl_2_; 100 μM acetosyringone]. The agrobacterium suspension was adjusted to an optical density at 600 nm (OD_600_) of 0.5 using a spectrophotometer and then infiltrated into three-week-old *N. benthamiana* leaves using a needleless syringe. For the simultaneous expression of two different proteins, equal volumes of agrobacterium suspensions were used.

### 2.4. Microsomal Fractionation

Microsomal fractionation was performed as previously described with a few changes [33]. Briefly, 1g of *N. benthamiana* leaf tissues expressing the target protein was harvested at 3 days post-inoculation (dpi) and ground in 4 mL of a lysis buffer (50 mM Tris-HCl, PH 7.4; 15 mM MgCl_2_; 10 mM KCl; 20% glycerol; 0.1% β-mercaptoethanol; one-quarter tablet of complete protease inhibitor (Roche, Shanghai, China)). After filtration through two layers of Miracloth, the solution was centrifuged at 3000 *g* for 10 min at 4 °C. The supernatant (S3) was separated into four equal volumes and then subjected to a second round of centrifugation at 100,000 *g* for 30 min at 4 °C, while the pellets (P3) were resuspended in 2 mL lysis buffer and then brought to the same volume as S3 with a lysis buffer. The resulting supernatants of the 100,000 *g* centrifugation (S100) were collected in a new centrifuge tube, while pellets containing crude membrane fractions (P100) were resuspended in the same volume of lysis buffer containing either 0.1 M Na_2_CO_3_ (PH 11.5), 4 M Urea, 8 M Urea, or 2% Triton X-100. After incubation for 30 min on ice, the samples were centrifuged again at 100,000 *g* for an additional 30 min at 4 °C. The resulting supernatants were collected, and the pellets were re-suspended in the same volume of lysis buffer. Equal volumes of the supernatant and suspension of each step were mixed with a 4×SDS loading buffer, boiled for 5 min at 95 °C, chilled on ice for 3 min, and then separated on 12% polyacrylamide gel after centrifugation at 10,000 rpm for 2 min at 4 °C.

### 2.5. Protein Purification from Plants

About 1 g of *N. benthamiana* leaf tissues expressing 10×His-MET_1-416_ was ground with 3 mL of binding buffer (50 mM Tris-HCl, pH7.4; 50 mM NaCl; 10% glycerol; 1% Triton X-100; 0.15% β-mercaptoethanol; 25 mM imidazole). Cell lysate was filtered through two layers of Miracloth and centrifugated at 3000 *g* for 10 min at 4 °C. The supernatant containing the target protein was transferred to a new tube and incubated with 100 μL of Ni-NTA agarose (Sangon Biotech, Shanghai, China) for 1 h at 4 °C. The supernatant containing Ni-NTA agarose was washed three times with a binding buffer on a chromatography column, and the target protein was dissolved in 500 μL of an elution buffer (50 mM Tris-HCl, pH 7.4; 50 mM NaCl; 10% glycerol; 250 mM imidazole).

### 2.6. Western Blotting

The proteins in polyacrylamide gel were transferred to a methanol-prewetted PVDF membrane by a Trans-Blot^®^ Turbo^TM^ transfer system (Bio-Rad, Shanghai, China) with a transfer buffer (25 mM Tris; 192 mM Glycine; 20% Methanol). After blocking for 1 h at room temperature or overnight at 4 °C in TBST (20 mM Tris, pH 7.5; 150 mM NaCl; 0.1% Tween 20) with 5% non-fat dry milk, the PVDF membrane was incubated in TBST with the appropriate detection antibodies for 1 h at room temperature. After washing six times with TBST, the PVDF membrane was incubated in TBST with proper secondary antibodies for 1 h at room temperature. The PVDF membrane was washed six times with TBST, wetted with an Immobilon Western chemiluminescent horseradish peroxidase (HRP) substrate solution (Millipore, Shanghai, China), and then visualized by a Tanon 5200 Chemiluminescent Imaging System (Tanon, Shanghai, China). The rabbit anti-GFP N-terminal domain (catalog no. G1544; Sigma-Aldrich), anti-mRFP (catalog no. 6g6; Chromotek), and Goat Anti-Rabbit IgG Peroxidase Conjugate antibodies (catalog no. DC03L; Sigma-Aldrich) were used at 1:5000 dilution.

### 2.7. Laser Scanning Confocal Microscopy

Confocal microscopy analysis was performed as described in [28,34]. In brief, a ~0.5 × 0.5 cm^2^ leaf patch, infiltrated with agrobacteria, was excised from a *N. benthamiana* seedling with a blade and monitored with Leica TCS SP8 laser scanning confocal microscopy (Leica, Germany). The YFP and mRFP were activated with 514 and 568 nm lasers, respectively. The sequential model was used for simultaneously recording two fluorescence signals.

### 2.8. Glutaraldehyde Cross-Linking Assay

In vivo glutaraldehyde cross-linking assay was performed as previously described with a few modifications [35]. In brief, *N. benthamiana* leaves expressing the target protein were harvested at 2 dpi, immerged in a cross-linking buffer (0.001% glutaraldehyde; 20 mM HEPES, pH 8.0), and then vacuumed for 10 min. After 1 h of incubation at room temperature, the leaves were rinsed by distilled H_2_O, dried with paper towel, and then ground into fine powder in liquid nitrogen, and homogenized in lysis buffer. The homogenate was clarified by centrifugation and then was used for Western blotting analysis.

For the treatment of purified proteins, different concentrations of glutaraldehyde were directly added into a protein solution. After 5 min of treatment, the reaction was stopped by adding 1/10 volume of 0.5 M Tris-HCl pH 8.0 solution. The reaction mixture was then boiled for 5 min at 95 °C, chilled on ice for 3 min, and then separated on 12% polyacrylamide gel after centrifugation at 10,000 rpm for 2 min at 4 °C.

### 2.9. Bioinformatics Analyses

The transmembrane motif was predicted using TMHMM Server v. 2.0 with default settings (https://services.healthtech.dtu.dk/service.php?TMHMM-2.0; accessed on 27 May 2021). Three-dimension models of the MET domain were predicted with Robetta and trRosetta with default settings [36,37]. The potential membrane-immersed amino acids for peripheral membrane proteins were predicted with DREAMM with default settings [38]. Structures were visualized and rendered with PyMOL (Available at: https://www.pymol.org/pymol.html; accessed on 27 May 2021).

## 3. Results

### 3.1. N-Terminal Part of PVX RdRp Is Peripherally Associated with ER

**Figure 1:**
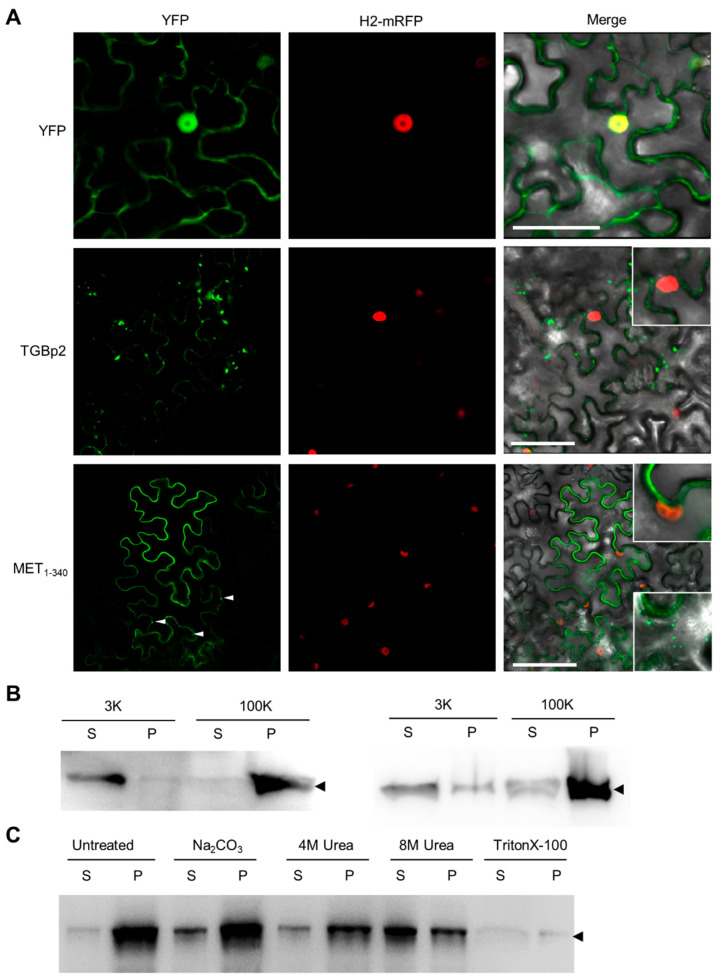
MET_1-340_ associated with ER. (**A**) Confocal microscopic photos of *N. benthamiana* epidermal cells expressing YFP, TGBp2-YFP, or MET_1-340_-YFP at 2 dpi. The nucleus is labeled by H2-mRFP. Insets show the typical nuclear signal. Scale bars = 50 μm. (**B**) Western blotting analysis pellets (P) and supernatants (S) of 3000 *g* (3K) and 100,000 *g* (100K) centrifugation by anti-GFP (left panel) or anti-mRFP antibodies (right panel). (**C**) Western blotting analysis showing the presence of MET_1-340_-YFP after the treatment of Na_2_CO_3_, urea, or TritonX-100 and 100,000 *g* centrifugation by anti-GFP antibodies.

Previously, we found that amino acids (aa) 1-340 of PVX RdRp that contains the N-terminal part of the MET domain (MET_1-340_) formed granules in the cell periphery [27]. To further analyze its subcellular localization, we transiently expressed a C-terminal YFP-tagged MET_1-340_ (MET_1-340_-YFP) in *N. benthamiana* epidermal cells. TGBp2-YFP and YFP were included as the membranous and cytosolic controls, respectively. Additionally, a C-terminal mRFP-tagged Histone H2A (H2-mRFP) was co-infiltrated to label the nucleus. YFP was located both in the cytosol and nuclei as diffusive fluorescence at 2 dpi, while TGBp2-YFP was exclusively located in the cell periphery as fluorescent foci of varied sizes at 2 dpi (Figure 1A). The fluorescence of MET_1-340_-YFP was located exclusively in the cell periphery as diffusive signals and granules in the cytoplasm at 2 dpi (Figure 1A). We found that the number of granular foci increased along with the expression time (Figure 1A), suggesting that MET_1-340_-YFP forms multimers via a slow folding process. These results suggested that MET_1-340_-YFP was associated with the endomembrane and may be able to form multimers.

Previous studies have shown that the replication of PVX takes place in the endoplasmic reticulum (ER), and the viral RdRps are colocalized with the ER [29,38]. We thus suspected that MET_1-340_ is colocalized with the ER and applied ultracentrifuge and chemical treatments to determine the interaction between MET_1-340_ and ER. We co-expressed MET_1-340_-YFP and a C-terminal mRFP-tagged ER maker BiP (BiP-mRFP) in *N. benthamiana* leaves via Agrobacterium-mediated infiltration. The leaves were harvested at 3 dpi for total protein extraction and microsomal fractionation analysis. The results showed that the majority of MET_1-340_-YFP was detected in the supernatant fraction of low-speed centrifugation and in the sediment of high-speed centrifugation (Figure 1B; left panel), suggesting that it is not associated with large intracellular membrane organelles, e.g., the nucleus and chloroplast, but associated with the ER. We found BiP-mRFP in the same fraction of both centrifugations (Figure 1B; right panel). The supernatant fraction of high-speed centrifugation was further treated with alkaline Na_2_CO_3_, mild chaotropic urea, or nonionic detergent Triton X-100 and analyzed by Western blotting with anti-YFP antibodies after a second round of high-speed centrifugation. We found that MET_1-340_-YFP could be dissolved by 8 M urea and Triton X-100, but not Na_2_CO_3_ and 4 M urea. Since the integral membrane protein was soluble only in Triton X-100, while the peripheral membrane protein could be extracted by alkaline Na_2_CO_3_ or chaotropic urea [39], these results indicated that MET_1-340_ was peripherally, but strongly, associated with the ER.

### 3.2. Prediction of Membrane-Immersed Amino Acids

**Figure 2:**
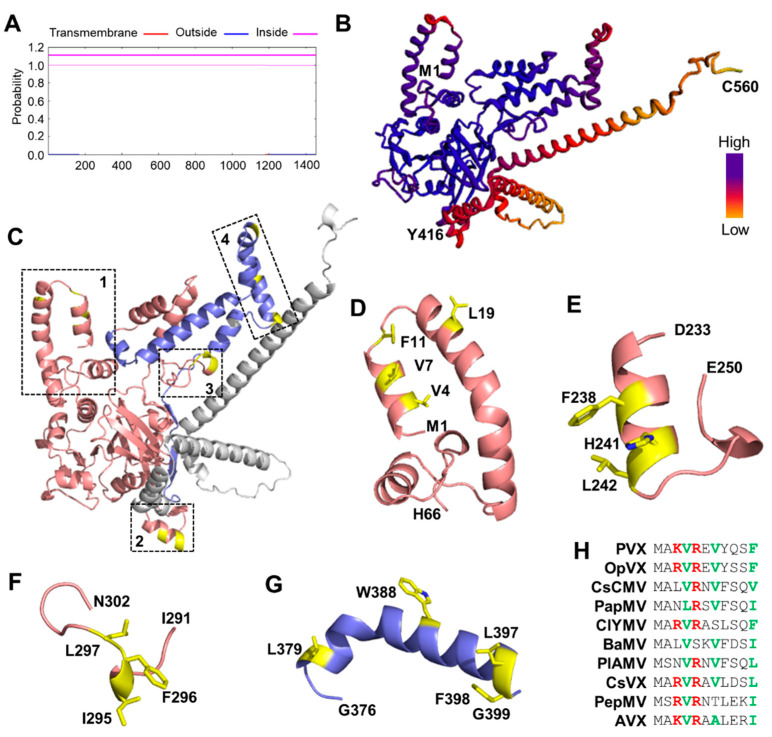
Bioinformatic analyses of the membrane-associated motif in the MET domain. (**A**) Prediction of transmembrane motif in PVX RdRp with TMHMM Server v. 2.0. (**B**) Ribbon representation of predicted PVX MET domain. The structure is colored based on confidence values (blue to yellow represents high to low confidence). The Met-1 (M1), Tyr-416 (Y416), and Cys-560 (C560) were labeled. (**C**) Cartoon representation of predicted PVX MET domain to show the locations of four predicted membrane-association motifs. Amino acids 1-340 of MET are colored in salmon, 341–416 aa are colored in light blue, 417–560 aa are colored in grey, and the putative hydrophobic residues are colored in yellow. (**D**–**G**) Cartoon representation of the four predicted membrane-association motifs. The start and stop positions of each motif are labeled and putative hydrophobic residues are also labeled and colored in yellow. (**H**) Multiple alignments of the foremost residues of representative potexviruses. Basic residues are in red and hydrophobic residues are in green. Viruses were selected based on the RdRp phylogeny of potexviruses [40]. OpVX, *opuntia virus X*; CsCMV, *cassava common mosaic virus*; PapMV, *papaya mosaic virus*; ClYMV, *clover yellow mosaic virus*; BaMV, *bamboo mosaic virus*; PlAMV, *plantago asiatica mosaic virus*; CsVX, *cactus virus X*; PepMV, *pepino mosaic virus*; AVX, *actinidia virus X*.

Similar to the PlAMV RdRp [31], no transmembrane motif was predicted by TMHMM within MET_1-340_ and even the entire RdRp of PVX (Figure 2A), indicating that MET_1-340_ may contact the ER via an unusual mechanism. Thus, we predicted the structure of varied lengths of the N-terminal part of the RdRp with Rebetta and trRosetta. We found that the N-terminal 1-416 aa (MET_1-416_) formed a compact domain with a high confidence score (Figure 2B). The potential membrane-immersed amino acids for peripheral membrane proteins were then predicted using the three-dimension model of MET_1-416_ with DREAMM [38]. Four regions with potential membrane-immersed amino acids were identified (Figure 2C): The first cluster of the potentially hydrophobic aa was located in the first two α- helixes (Val-4, Val-7, Phe-11, and Leu-19; Figure 2D), the second cluster of the potentially hydrophobic aa was located between 238–242 aa (Phe-238, His-241, and Leu-242; Figure 2E), the third cluster of the potentially hydrophobic aa was located in a loop region between 291–302 aa (Phe-296, Ile-295, Leu-297; Figure 2F), while the fourth cluster of the potentially hydrophobic aa was located in an α-helix between 376–399 aa (Leu-379, Trp-388, Leu-397, Phe-398; Figure 2G), which correspond to the previously described proline-kinked amphipathic α-helix in PlAMV [31]. Moreover, the predicted membrane-immersed hydrophobic residues in the first α-helix were functionally conserved in most potexviruses (Figure 2H).

### 3.3. The First α-Helix Is Essential for Membrane Targeting

**Figure 3:**
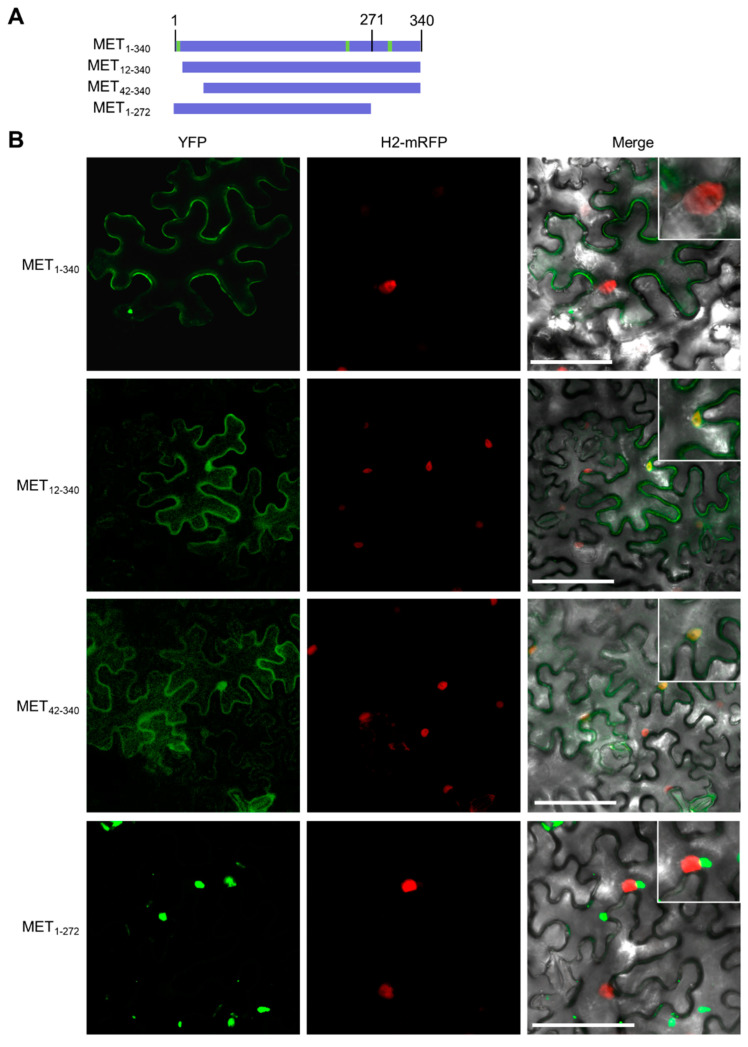
The first α-helix is required for membrane-association. (**A**) Illustration of MET_1-340_ mutants. The three putative membrane-association motifs in MET_1-340_ are highlighted in yellow. (**B**) Confocal microscopic photos of *N. benthamiana* epidermal cells expressing MET_1-340_-YFP, MET_12-340_-YFP, MET_42-340_-YFP, and MET_1-272_-YFP at 2 dpi. The nucleus was labeled by H2-mRFP. Insets show the typical nuclear signal. Scale bars = 50 μm.

Three truncated mutants were constructed to identify the bona fide membrane-associated motif in MET_1-340_ (Figure 3A). The first α-helix was deleted in MET_12-340_, both the first and second α-helixes were deleted in MET_42-340_, while the protrude C-termini of MET_1-340_ harboring the third potential membrane-associated motif was deleted in MET_1-271_. These mutants were transiently expressed in *N. benthamiana* epidermal cells as C-terminal YFP-tagged recombinant proteins via agroinfiltration. At 2 dpi, the subcellular localization was observed under a confocal microscope. The results showed that the fluorescence of both MET_12-340_-YFP and MET_42-340_-YFP was observed in the nuclei, while the fluorescence of MET_1-271_-YFP was found exclusively in the cytosol as granules (Figure 3B). These results indicated that the first α-helix might be essential for MET_1-340_ to anchor on the cellular membrane, while the second and third potentially hydrophobic regions were not bona fide membrane-associated motifs. We noticed that the granular fluorescent foci formed by MET_1-340_-YFP were almost completely dismissed in MET_12-340_-YFP and MET_42-340_-YFP (Figure 3B), suggesting that the first α-helix might also be involved in multimerization.

### 3.4. Hydrophobic Residues in the First α-Helix Are Required for Multimerization

**Figure 4:**
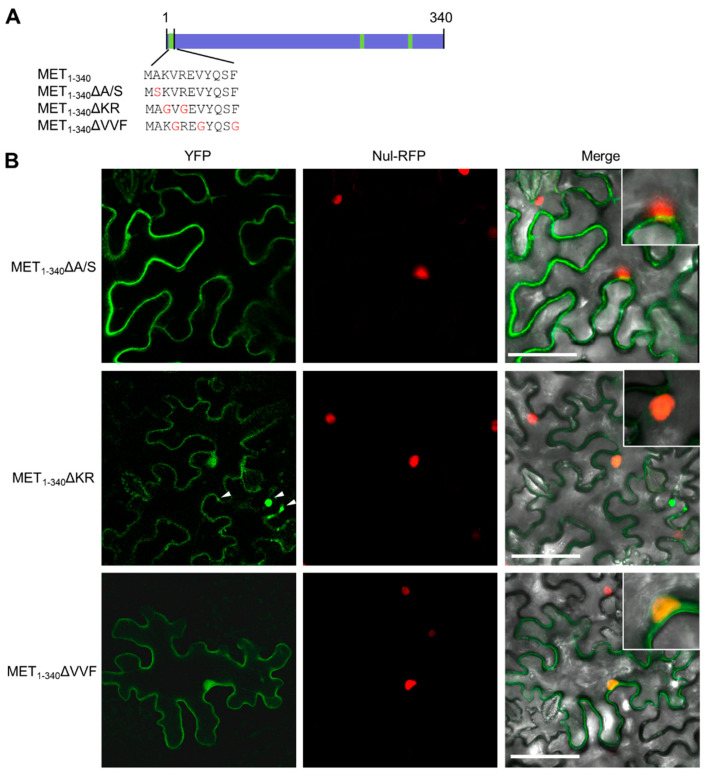
Hydrophobic residues in the first α-helix are required for multimerization. (**A**) Illustration of point mutants of MET_1-340_. The three putative membrane-association motifs in MET_1-340_ are highlighted in green. (**B**) Confocal microscopic photos of *N. benthamiana* epidermal cells expressing MET_1-340_ΔA/S-YFP, MET_1-340_ΔKR-YFP, and MET_1-340_ΔVVF-YFP at 2 dpi. The nucleus was labeled by H2-mRFP. Insets show the typical nuclear signal. Scale bars = 50 μm.

Point mutations were introduced into the first N-terminal α-helix to further understand its function in membrane association and multimerization (Figure 4A). The Ala-2 residue after Met-1 was changed into Ser in MET_1-340_ΔA/S, the two basic resides (Lys-3 and Arg-5) in the first α-helix were replaced with Gly in MET_1-340_ΔKR, while the three phydrophobic residues (Val-4, Val-7, and Phe-11) in the first α-helix were changed into Gly in MET_1-340_ΔVVF. We then transiently expressed these mutants in *N. benthamiana* epidermal cells as C-terminal YFP-tagged recombinant proteins. The subcellular localization was observed at 2 dpi by a confocal microscope. The results showed that replacing the basic residues in the first α-helix of MET_1-340_ abolished its membrane association but retained its multimerization ability, while replacing Ala-2 with Ser had no obvious influence to the membrane association and multimerization of MET_1-340_ (Figure 4B). Changing Val-4, Val-7, and Phe-11 to Gly resulted in the complete loss of membrane association and multimerization (Figure 4B), consisting with the natural occurrence of Ser-2 in PlAMV (Figure 2H). Together, these results further confirmed that phydrophobic residues in the first α-helix of MET_1-340_ might be essential for membrane targeting and multimerization.

### 3.5. MET Forms Multimers

**Figure 5:**
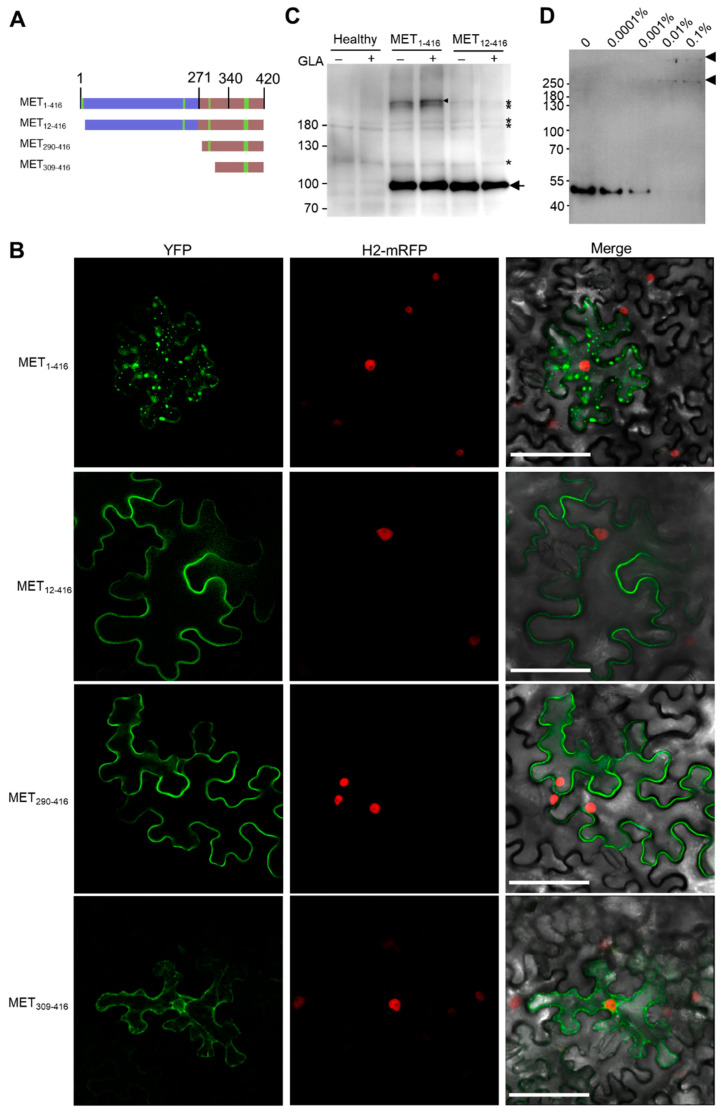
MET domain of PVX RdRp forms multimers. (**A**) Illustration of MET1-416 mutants. The four putative membrane-association motifs are highlighted in yellow. (**B**) Confocal microscopic photos of *N. benthamiana* epidermal cells expressing MET_1-416_-YFP, MET_12-416_-YFP, MET_290-416_-YFP, and MET_309-416_-YFP at 2 dpi. (**C**) Western blotting analyses of MET_1-416_-YFP and MET_12-416_-YFP after glutaraldehyde treatment. (**D**) Western blotting analyses of purified 10×His-MET_1-416_ that was treated by different concentrations of glutaraldehyde.

A previous study discovered an amphipathic α-helix between 363 to 380 aa of the PlAMV MET domain [31]. The corresponding α-helix of the PVX MET domain (376–399 aa) was also predicted to be potentially hydrophobic (Figure 2G). We thus suspected that both the first α-helix and this α-helix regulates membrane association and multimerization of the MET domain. Amino acids 1-416 of PVX RdRp (MET_1-416_) form a compact domain in the predictive model with a high confidence score (Figure 2A). We analyzed the subcellular localization of MET_1-416_ as the C-terminal YFP-tagged recombinant protein (MET_1-416_-YFP) in *N. benthamiana* epidemical cells. Confocal microscopy observation showed that MET_1-416_-YFP was located in the cytosol as granules at 2 dpi (Figure 5B), suggesting that MET_1-416_ is associated with the endomembrane, and the C-terminal 341–416 aa can add its multimerization. We further produced three truncated mutants, namely MET_12-416_, MET_290-416_, and MET_309-416_. MET_12-416_ lost the first α-helix, MET_290-416_ lost the first hydrophobic α-helix and the second putative membrane motif, while MET_309-420_ contained only the fourth putative amphipathic α-helix (Figure 5A). These three mutants were transiently expressed in *N. benthamiana* epidermal cells as C-terminal YFP-tagged recombinant proteins. Confocal microscope results showed that all of them were located in the cell periphery and no fluorescent signal was recorded in the nucleus at 2 dpi (Figure 5B), further confirming that the fourth amphipathic α-helix is a bona fide membrane-associated motif [31]. No fluorescent granule was observed in *N. benthamiana* epidermal cells expressing MET_12-420_-YFP, MET_290-420_-YFP, or MET_309-416_-YFP (Figure 5B), suggesting that the first α-helix is essential for multimerization.

An in vivo glutaraldehyde cross-linking assay was performed to compare the multimerization of MET_1-416_-YFP, and MET_12-416_-YFP. Therefore, *N. benthamiana* epidermal cells expressing MET_1-416_-YFP or MET_12-420_-YFP were treated with 0.001% glutaraldehyde and analyzed by Western blotting using anti-GFP antibodies. The results showed that the treatment of glutaraldehyde resulted in the appearance of a band above the highest band of the protein ladder (180 kDa; Figure 5C), suggesting that MET_1-416_ forms multimers. In contrast, glutaraldehyde treatment of MET_12-420_-YFP did not result in a corresponding band (Figure 5C), confirming that the first α-helix is essential for multimerization. To further analyze the multimeric status of MET, we expressed a 10×Histidine-tagged MET_1-416_ (10×His-MET_1-416_) in *N. benthamiana*. After Ni-NTA purification, 10×His-MET_1-416_ was treated with different concentrations of glutaraldehyde, separated by SDS-PAGE, and analyzed by Western blotting. The results showed that the band of about 50 kDa, which represents monomer 10×His-MET_1-416_, decreased along with the increment of glutaraldehyde concentration; in the meantime, two bands, one band slightly higher than 250 kDa and another one with a much higher molecular weight, were detected after the treatment of 0.01% and 0.1% glutaraldehyde (Figure 5D). These results suggested that the MET_1-416_ might form hexamers and/or even larger multimers, e.g., dodecers.

## 4. Discussion

The replication of +ssRNA viruses takes place in virus-induced membranous organelles to achieve maximum efficiency and to escape host dsRNA-based immunity. The replication-associated proteins of different +ssRNA viruses adopt varied strategies to target and remodel the endomembrane. For PlAMV, a proline-kinked amphipathic α-helix at the C-terminal of the MET domain was recently found to be required for its membrane association [30], which was further confirmed by our results. A fragment of MET without the proline-kinked amphipathic α-helix was still located in the cytosol and colocalized with the ER, suggesting that there were additional hydrophobic motifs in the MET domain. Bioinformatic analyses revealed three additional putative hydrophobic motifs in the MET domain (Figure 2). Mutagenesis and subcellular localization analyses showed that the first α-helix was a bona fide membrane-association motif (Figure 3). These results suggested that the MET domain was associated with the endomembrane, most possibly the ER, via at least two hydrophobic motifs. Similar strategies were observed in other +ssRNA viruses, e.g., the membrane association of 1a protein of BMV also requires an amphipathic α-helix and one or more current unknown hydrophobic motifs [17,39]. Instead, nsP1 of alphaviruses utilizes two membrane-binding spikes with hydrophobic tips to pierce the endomembrane [15,20]. Therefore, it is possible that varied +ssRNA viruses adopted different membrane-association strategies, and MET of potexviruses may have evolved a unique mechanism for membrane association.

Recent cryo-EM studies have shown that protein A of FHV and nsp1 of alphaviruses, both of which have methyltransferase- and RNA-capping activities, form crown complexes on membranes [19,21,22]. We also found that MET of PVX formed multimers on the ER (Figure 5B). Glutaraldehyde cross-linking assay showed that the MET domain formed at least pentamers (Figure 5D). Thus, it is possible that MET also forms multimers in VROs. It has been proposed that form multimers facilitate the capping of genomic RNA [19], a process essential for the replication of viral RNA with a cap structure on the 5′-termini. Therefore, it is possible that the forming of multimers may be a common feature and requirement of +ssRNA methyltransferases. Nevertheless, further biochemical and structural analyses are needed to determine the multimeric status of the potexviral MET domain and the function of multimerization in viral replication, and whether it also forms crown-like structures. The most significant finding of our study was that the first α-helix was required not only for membrane association, but also for its multimerization. Previous studies have shown that the membrane association of alphaviral nsp1 is coupled with its multimerization [15,20]. The same could be true for PVX MET. However, further studies are needed to illustrate this possibility. Although the structures of VROs have been studied in detail for some animal RNA viruses [15,20,22], such studies for plant RNA viruses are still lacking. For PVX, the methyltransferase, helicase, and replicase are located in the same polypeptide. How these domains are arranged in the VROs, and how they regulate different processes of genome replication, e.g., capping, dsRNA unwinding, replication, and RNA release, are worth studying in the future.

## 5. Conclusions

This study analyzed putative hydrophobic motifs in the MET domain of PVX and found that the first α-helix is a bona fide membrane-association motif. Moreover, we confirmed that this α-helix was essential for the multimerization of the MET domain. We also analyzed the multimeric status of the MET domain using glutaraldehyde cross-linking assay. These results shed new light on the membrane association and multimerization of the MET domain of PVX and possibly other potexviruses as well.

## Data Availability

All data have been included in the text or as supplementary files.

## Supplementary Materials

The following supporting information can be downloaded at: https://www.mdpi.com/article/10.3390/v14091907/s1, Table S1: primers used in the present study.
